# Determination of the Degree of Crystallinity of Poly(2-methyl-2-oxazoline)

**DOI:** 10.3390/polym13244356

**Published:** 2021-12-13

**Authors:** Evgeniy M. Chistyakov, Sergey N. Filatov, Elena A. Sulyanova, Vladimir V. Volkov

**Affiliations:** 1 Mendeleev University of Chemical Technology of Russia, 125047 Moscow, Russia; Filatovsn@list.ru; 2 Shubnikov Institute of Crystallography, Federal Scientific Research Centre “Crystallography and Photonics”, Russian Academy of Sciences, 119333 Moscow, Russia; sulynova@gmail.com (E.A.S.); volkicras@mail.ru (V.V.V.)

**Keywords:** oxazolines, polyoxazolines, 2-methyl-2-oxazoline, poly(2-methyl-2-oxazoline), crystallinity

## Abstract

A new method for purification of 2-methyl-2-oxazoline using citric acid was developed and living cationic ring-opening polymerization of 2-methyl-2-oxazoline was carried out. Polymerization was conducted in acetonitrile using benzyl chloride—boron trifluoride etherate initiating system. According to DSC data, the temperature range of melting of the crystalline phase of the resulting polymer was 95–180 °C. According to small-angle X-ray scattering and wide-angle X-ray diffraction data, the degree of crystallinity of the polymer was 12%. Upon cooling of the polymer melt, the polymer became amorphous. Using thermogravimetric analysis, it was found that the thermal destruction of poly(2-methyl-2-oxazoline) started above 209 °C.

## 1. Introduction

Currently, polymeric materials based on polymers that have not been popular in the past are employed more and more often to improve the living standards of people and to develop advanced technologies. Some of these polymers were considered forgotten. Today, such polymers are finding increasing practical use and are becoming the objects of numerous studies. For example, highly efficient lithium ion batteries are produced using chitosan, a natural renewable polymer [1]. Phosphazene-containing polybenzoxazines and epoxy resins serve as binders for non-combustible polymer composites [2,3] and condensation of some aryloxyphosphazenes results in the formation of thermally stable matrix polymers [4,5]. Silsesquioxane–siloxanes are excellent modifying agents for dental materials [6]. The vinyl sulfide polymer can efficiently extract Pd(II) from chloride solutions [7]. Quite a few electrically conductive polymers are used in electronics [8]. Poly(vinyldiphenylphosphine) can serve for the synthesis of various olefins [9]. In the field of biomedical research, the application of specific polymers such as polylactic acid [10], polyaniline, and polypyrrole [11] was revived. Poly-2-oxazolines are also among such polymers. They were used to prepare pH-sensitive and thermosensitive gels, drug delivery agents, shells for magnetic particles, antibacterial films and coatings, liposomes, materials for cell engineering, and for many other purposes [12,13,14,15,16,17,18,19,20,21,22,23,24,25]. Nevertheless, poly-2-oxazolines have been poorly studied as yet. It is known that poly(2-alkyl-2-oxazolines) containing more than two carbon atoms in the alkyl chain are crystalline [26,27,28]. Polymers with methyl and ethyl side groups are considered to be amorphous [29,30]. However, we found that the degree of crystallinity of poly(2-methyl-2-oxazoline) can reach 12%. This should be taken into account for polymer processing, manufacture of polymer products, and evaluation of the physicochemical and physicomechanical properties of polymer-based materials.

## 2. Materials and Methods

### 2.1. Materials

Dichloromethane (anhydrous, ≥99.8%), acetonitrile (anhydrous, 99.8%), monoethanolamine (purified by redistillation, ≥99.5%), diethyl ether (anhydrous, ≥99.7%), benzyl chloride (ReagentPlus^®^, 99%), boron trifluoride etherate (purified by redistillation, ≥46.5% BF_3_ basis), citric acid (ACS reagent, ≥99.5%), zinc chloride (anhydrous, free-flowing, Redi-Dri™, reagent grade, ≥98%), potassium carbonate (ACS reagent, ≥99.0%), and magnesium sulfate (anhydrous, ReagentPlus^®^, ≥99.5%) were Sigma-Aldrich chemicals, Saint Louis, MO, USA. The chemicals were used as received.

### 2.2. Synthesis of 2-Methyl-2-oxazoline

Anhydrous zinc chloride (6.82 g, 0.05 mol) was poured into a 250-mL one-necked round-bottom flask with a magnetic stir bar. Acetonitrile (70 mL, 1.34 mol) and monoethanolamine (100 mL, 1.66 mol) were added. A reflux condenser equipped with an argon inlet (argon flow rate of 10 mL/min) was inserted into the neck of the flask. The reaction mixture was heated to 82 °C with stirring. The synthesis was carried out until the evolution of ammonia from the outlet tube ceased (determined using litmus paper strips). Crude 2-methyl-2-oxazoline was isolated from the reaction mixture by distillation to collect the fraction boiling at 108–112 °C. The yield of the monomer was 82 g (72% yield of the theoretical amount). For purification, citric acid (2 g) was dissolved in the monomer, the solution was kept for 2 h and then poured with stirring into dichloromethane (150 mL). After the formation of a precipitate, the liquid phase was transferred into a 250 mL flat-bottom flask and potassium carbonate (3 g) and magnesium sulfate (3 g) were added. The flask was tightly closed, the dispersion was stirred for 4 h, then the precipitate was filtered off, and the liquid phase was distilled to collect the fraction boiling at 110 °C. The yield of pure 2-methyl-2-oxazoline was 92 wt % relative to the starting crude material. The refractive index of the monomer was n_20_D = 1.430.

### 2.3. Synthesis of Poly(2-methyl-2-oxazoline)

2-methyl-2-oxazoline (2 g, 0.023 mol) and acetonitrile (5 mL) were loaded into a 15 mL round-bottom flask, which was equipped with a reflux condenser and a magnetic stir bar. Then boron trifluoride etherate (0.028 g, 0.0002 mol) and benzyl chloride (0.025 g, 0.0002 mol) were added with stirring. The reaction was conducted under argon flow (10 mL/min) without stirring for 24 h at the solvent boiling point. Then, the reaction mixture was cooled down to room temperature and acetonitrile (5 mL) and distilled water (0.1 mL) were added. The solution was stirred for 20 min and poured into diethyl ether. This resulted in the separation of a yellow resinous liquid, which was dried in vacuo at 60 °C to a constant weight. The polymer was obtained as a foamed solid in a yield of 1.2 g (65%).

The polymerization was stopped before the monomer conversion was complete to restrict the polymer molecular weight. Otherwise, it was impossible to prepare samples for small-angle X-ray scattering measurements because of too high viscosity.

### 2.4. Sample Preparation for Determining the Degree of Crystallinity of the Polymer

A portion (0.1 g) of the synthesized polymer was dissolved in dichloromethane (0.5 mL), the solution was taken into a syringe and deposited dropwise onto a heated (35 °C) Teflon substrate, with the solvent being evaporated between drops. The sample diameter was controlled within 5–7 mm. After the whole solution had been deposited, the substrate was cooled down and placed into a vacuum oven, in which the sample was kept for 5 h at room temperature and a residual pressure of 0.5 bar. After that, the pressure was decreased to 0.1 bar and the sample was kept for 5 h, then the temperature was raised first to 35 °C (10 h) and then to 45 °C (10 h). The dry film was separated from the substrate and its thickness was measured to be ~0.1 mm.

### 2.5. Methods

Differential scanning calorimetry (DSC) measurements were performed using a NETZSCH STA 449F1 instrument (Erich NETZSCH GmbH & Co. Holding KG, Selb, Germany). A ~10 mg portion of the polymer was placed into an aluminum crucible, which was then pressurized. The crucible was placed into the measuring cell. A reference crucible was placed into the second cell. The heating and cooling mode (10 °C min^–1^) was specified with the Proteus Analysis software. Argon was used as a purge gas. The results were processed using the Proteus^®^ software.

Thermogravimetric analysis (TGA) was carried out on a Derivatograph-C instrument (MOM SZERVIZ KFT., Budapest, Hungary) under argon, using ~10 mg samples and heating rate of 10 °C min^–1^ (the TGA curve is presented in the Supplementary Materials).

^1^H and ^13^C NMR spectra were recorded on a Bruker AV-400 spectrometer, Bruker Corporation, Billerica, MA, USA (the spectra are given in the Supplementary Materials).

Gel permeation chromatography (GPC) was performed using a Waters system comprising a Waters 1525 gradient pump, a Waters 1500 column thermostat, a Waters 2414 refractive index detector, and a Waters 2707 cooled autosampler (Waters Corporation, Milford, MA, USA), and a 300 × 7.8 mm TSKgel-G-Oligo-PW chromatographic column filled with a hydroxylated polymethacrylate gel (7 μm) (Tosoh Corporation, Shiba, Minato-ku, Tokyo, Japan). Polyethylene glycol standards with peak-average molecular weights (Mp) of 106, 430, 1030, 2130, 3450, and 6530 Da (PSS) were used. A 0.1 M aqueous solution of NaNO_3_ served as the mobile phase. The concentration of the test aqueous solution was 2 mg/mL. The GPC curve and the obtained characteristics of the polymer are given in the Supplementary Materials.

The small-angle X-ray scattering intensities were measured with the AMUR-K automated small-angle X-ray diffractometer (FRC Crystallography and Photonics, Moscow, Russia) with a linear position-sensitive detector (3300 channels) at a fixed wavelength λ = 0.1542 nm (CuKα line of a fine-focus tube, a pyrolytic graphite monochromator) and a Kratky collimation system. The X-ray beam cross-section was 0.2 by 8 mm and the angular range was 0.07° < 2θ < 7.05°. The sample was placed into a vacuum chamber with a sample to detector distance of 700 mm. The time of the measurement was 1 h. The experimental data were normalized to the intensity of the incident beam, after which a correction for collimation distortions was applied [31]. Crystallite sizes were estimated using the Debye–Scherrer formula [32]

$$L=\frac{K\cdot \lambda }{\Delta (2\theta )\cdot \cos\theta }$$

where Δ(2θ) is full width at half maximum, θ is the Bragg angle, *K* is the Scherrer constant in the range 0.89–1.2, depending on the outer shape and packing type of the crystalline grain. The calculations were performed using the PEAK program from the ATSAS package [33]. Before processing the scattering curves, noise filtering was performed using the nonparametric adaptive smoothing program. 

The wide-angle diffraction pattern up to 2θ = 40° was measured on the single-crystal X-ray diffractometer Xcalibur S Agilent Technologies equipped with the Kappa geometry KM-4 goniometer (Oxford Diffraction Limited, Abingdon, Oxfordshire, UK). The X-ray source was Mo Kα, λ = 0.71073 Å, with the beam cross-section 0.5 × 0.5 mm. Two-dimensional CCD detector Sapphire S3 (2048 × 2048 pixels) was used with the distance to the sample 41.5 mm. The measurements were carried out with the rotation of the sample by 360 degrees for better averaging.

The lattice parameters of the sample were calculated with DICVOL14 program [34]. Then the space group *P*2/*m* was chosen during full-profile analysis using FullProf software [35]. The shape of MoK_α_ peaks of the sample was adequately described by the pseudo-Voigt function using Thompson–Cox–Hastings approximation [36]. The background was adjusted with the 4th degree polynomial. Le Bail fitting [37] with constant scale factor was performed with the following parameters having been refined: scale factor, the zero-point shift, unit cell parameter, pseudo-Voigt peak profile parameters, and the overall isotropic temperature factor.

## 3. Results and Discussion

**Figure 1:**
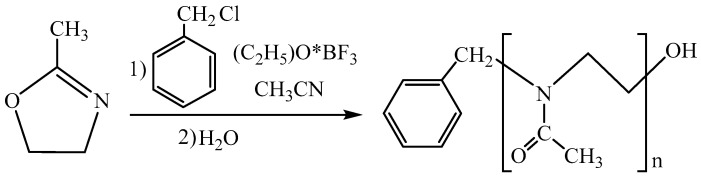
2-methyl-2-oxazoline polymerization scheme.

Poly(2-methyl-2-oxazoline) was synthesized by cationic ring-opening polymerization according to the scheme depicted in Figure 1. The initiating system comprised two components, benzyl chloride and boron trifluoride etherate.

The monomer for this reaction was additionally purified by a specially developed procedure using citric acid to remove all basic impurities.

**Figure 2:**
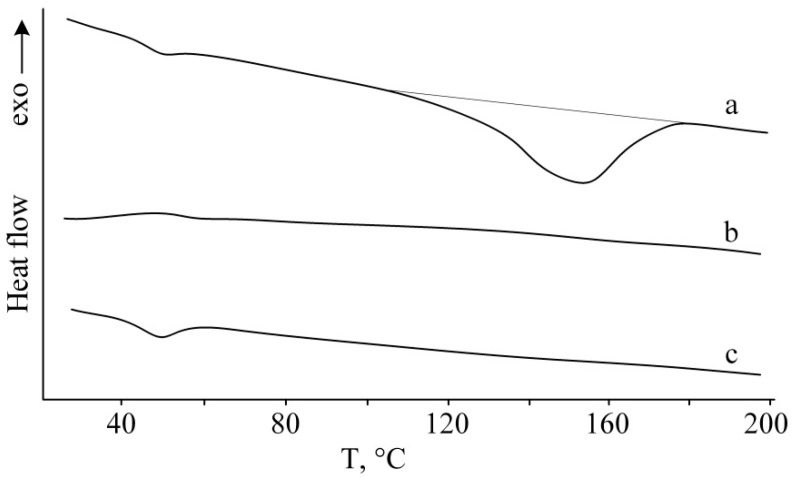
DSC curves of poly(2-methyl-2-oxazoline): first heating (**a**), cooling (**b**), and repeated heating (**c**).

A differential scanning calorimetry study of the resulting polymer demonstrated that, apart from the heat capacity step at 40–60 °C corresponding to the glass transition temperature, the curve shows a broad endotherm in the range of 95–180 °C (Figure 2a).

It was suggested that the peak corresponds to the melting of the crystalline phase of poly(2-methyl-2-oxazoline). This hypothesis was verified by measuring the intensity of small-angle X-ray scattering of a polymer film produced by a solution method.

**Figure 3:**
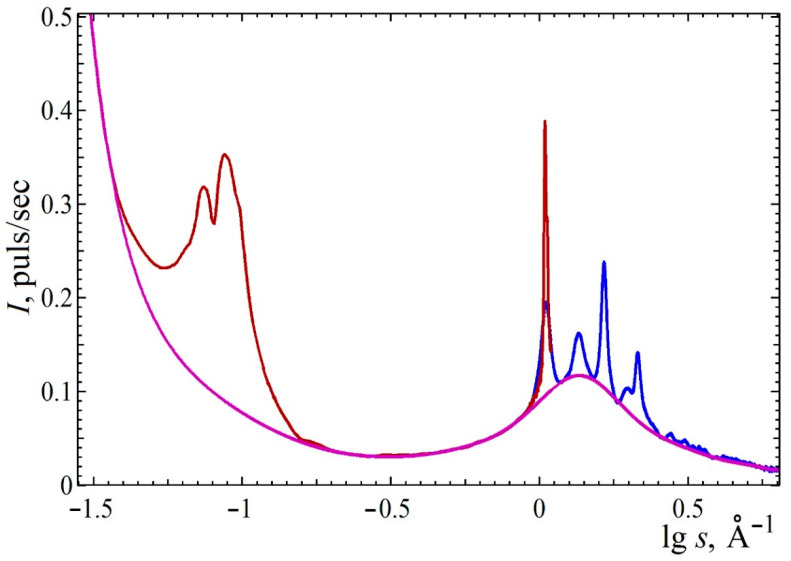
Experimental scattering intensity from poly(2-methyl-2-oxazoline). Red line: small-angle scattering part; blue: wide-angle diffraction pattern; magenta: baseline corresponding to the scattering from the disordered phase.

The radial scattering functions measured on the AMUR-K small-angle diffractometer and Xcalibur S diffractometer are shown in Figure 3. To determine the packing parameters and the degree of crystallinity, the diffuse scattering background line was calculated as an 18th degree approximating polynomial passing through the selected background regions of the scattering curve. To ensure the monotonicity of the background curve, the calculations were performed in a double logarithmic scale followed by conversion of the result to the original scale. The degree of the polynomial was chosen under the condition of the minimum integral curvature of the background line. The obtained baseline and the result of its subtraction from the experimental data are shown in Figure 3. After accounting for the diffuse background, the areas under the peak at 2θ = 6.8° in both measurements coincided with an accuracy of 12%. The larger width of the peaks on the wide-angle part of the curve is due to the large beam cross section in the Xcalibur S diffractometer, which was set to increase the signal-to-noise ratio.

As a result, two groups of diffraction peaks corresponding to two systems of crystallites with interplanar spacings, shown in Table 1, were detected. The resulting baseline and the result of its subtraction from the experimental data are shown in Figure 3. The angular scale is expressed in the scattering vector moduli $|S|=\frac{4\pi \cdot \sin\theta }{\lambda }$, where 2θ is the scattering angle in radians.

The group of peaks with large interplanar spacings corresponds to the packing of polymer globules with interplanar distances of about 70 Å; peaks at large scattering angles correspond to the packing of polymer chains. The fact that the crystallite sizes of about 450 Å are much larger than the interplanar distance in the globule packing, it may be proposed that the crystalline phase does not correlate with the inner structure of the globules.

Background scattering corresponds to scattering from the disordered phase of the sample and atomic scattering. There are several approaches to calculating the degree of crystallinity ([38], Chapter 3). The simplest approach is to calculate the ratio of the areas under the diffraction peaks after subtracting the amorphous background curve to the background area in the maximum available range of angular measurements. This approximation is independent of the lattice parameters and is given under the assumption that the shape of the amorphous phase does not change with changing amorphous content. The difference between the absorption coefficients of crystalline and amorphous polymers is assumed to be the same. To more adequately account for the atomic scattering background, the atomic background curve was extrapolated to an angle of 2θ = 24° using the atomic scattering curves published in [39]. The estimate gives the degree of crystallinity about 0.12. In these calculations, we did not take into account the low-angle group of peaks from the packing of polymer globules at 2θ = 0.55°, combining it with the background curve.

**Figure 4:**
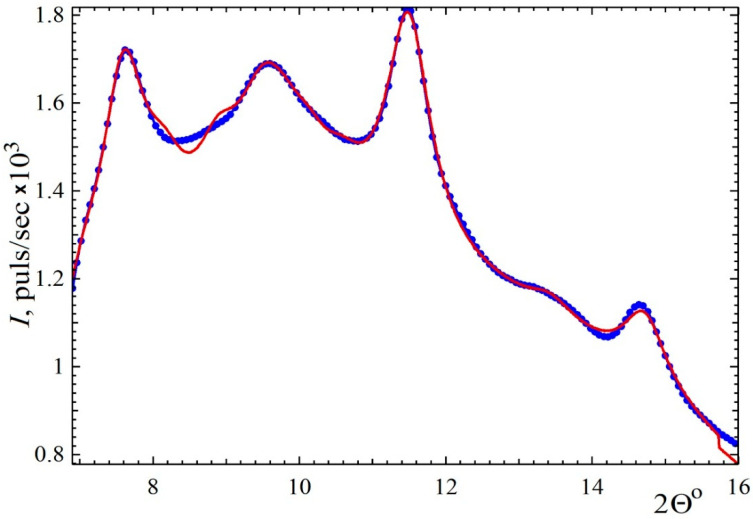
Result of cell parameter determination using the FullProf package. Dots: experimental data; line: fit by monoclinic Pm/2 model.

Because of the small number of peaks in the wide-angle region, the identification of the spatial group is ambiguous. A full-profile analysis showed the best agreement between the theoretical scattering and the experimental one (presented in Figure 4) for the monoclinic *P*2/*m* group with cell parameters a = 10.75(2), b = 7.512(9), c = 5.79(1) Å, and β = 92.56(9)°. χ^2^ = 1.5.

It is noteworthy that the cooling of the polymer heated above the melting point of the crystalline phase is not accompanied by sample crystallization (Figure 2b). The DSC curve shows only a heat capacity step at 40–60 °C, corresponding to the polymer glass transition. During repeated heating of the sample, devitrification of poly(2-methyl-2-oxazoline) takes place at the same temperature (40–60 °C); however, no melting peak for the crystalline phase is present in the curve (Figure 2c). This implies that ordering of the polymer structure occurs only during the polymer synthesis, which is apparently due to the coordination effect of the initiating system.

## 4. Conclusions

Poly(2-methyl-2-oxazoline) demonstrated a relatively high degree of crystallinity when the sample was prepared by casting from solution. However, like isotactic polystyrene, poly(2-methyl-2-oxazoline) irreversibly transforms into an amorphous state after melting and does not crystallize during subsequent cooling. Nevertheless, the crystalline phase of the polymer can still be preserved by polymer processing from solutions. In any case, the glass transition temperature of poly(2-methyl-2-oxazoline) is approximately 50 °C and the thermal stability is about 209 °C, which should be taken into account for the heat treatment and application of this polymer.

## Figures and Tables

**Table 1:** Parameters of leading diffraction peaks of crystallite systems. The crystalline sizes for low-resolution wide-angle diffractometry are not presented due to instrumental broadening.

Peak Position, Å^−1^	Interplanar Bragg Spacing, Å	Coherence Length (Size of the Crystalline Grain) Found by the Debye–Scherer Formula, Å at *K* = 1.11
0.0743 ± 0.0003	85 ± 1	-
0.0885 ± 0.0003	71 ± 1	-
0.0981 ± 0.0005	64 ± 2	-
1.0424 ± 0.0003	6.03 ± 0.02	430 ± 30
1.0574 ± 0.0003	5.94 ± 0.02	480 ± 40
1.3502 ± 0.0003	4.64 ± 0.02	-

## Supplementary Materials

The following are available online at https://www.mdpi.com/article/10.3390/polym13244356/s1, Figure S1: ^1^H NMR spectrum 2-methyl-2-oxazoline, Figure S2: ^13^C NMR spectrum 2-methyl-2-oxazoline, Figure S3: ^1^H NMR spectrum poly(2-methyl-2-oxazoline), Figure S4: ^13^C NMR spectrum poly(2-methyl-2-oxazoline), Figure S5: GPC curve of the synthesized poly(2-methyl-2-oxazoline), Figure S6: TGA curve poly(2-methyl-2-oxazoline), Table S1: Molecular weight characteristics of the synthesized poly(2-methyl-2-oxazoline).
