# Supplementary material for: Determination of the Degree of Crystallinity of Poly(2-methyl-2-oxazoline)
Source: Polymers (Basel). 2021 Dec 13;13(24):4356. doi: 10.3390/polym13244356 (PMC8704864; doi:10.3390/polym13244356)
Supplement: Supplementary file 1 [file polymers-13-04356-s001.zip › polymers-1385702-supplementary.pdf]

Supplementary Materials

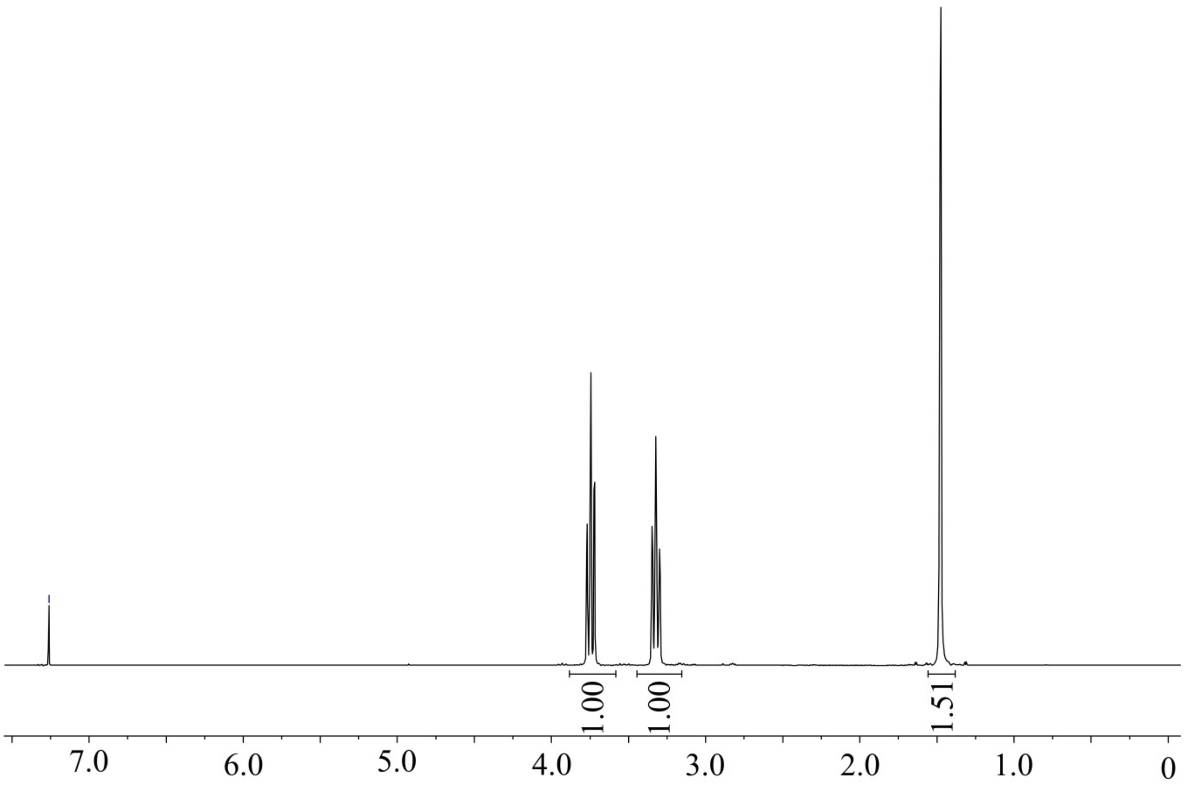

Fig. S1 – ^{1}H NMR spectrum 2-methyl-2-oxazoline.

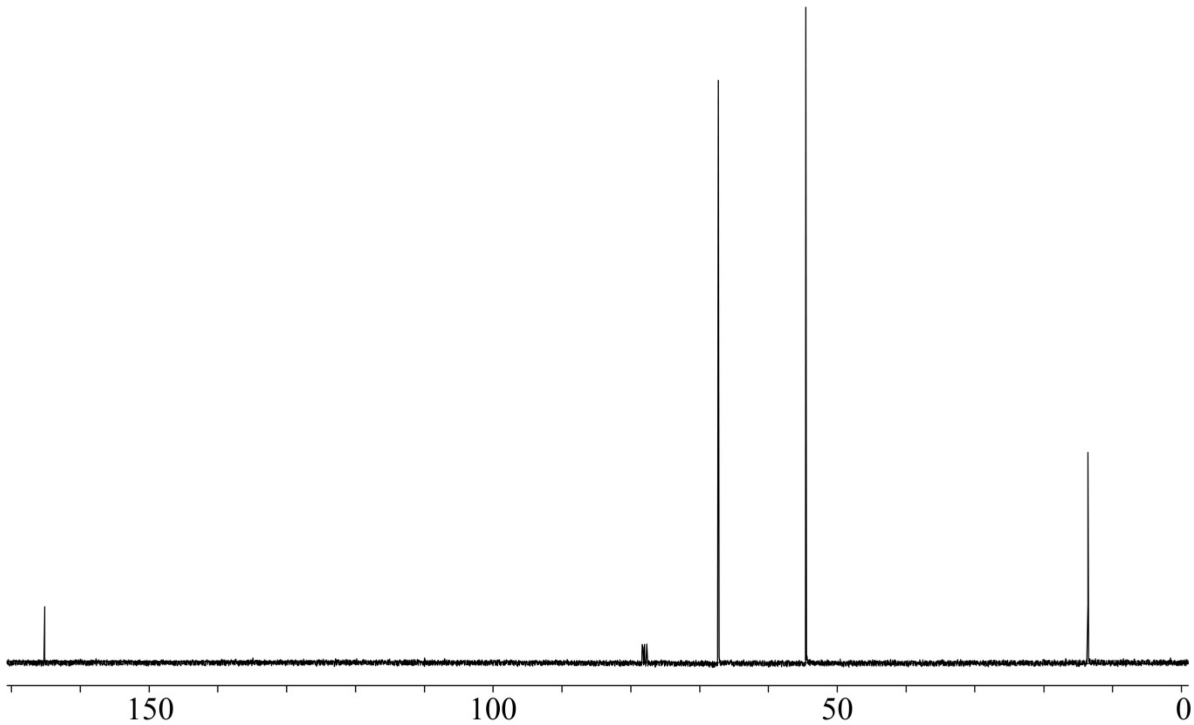

Fig. S2 – ^{13}C NMR spectrum 2-methyl-2-oxazoline.

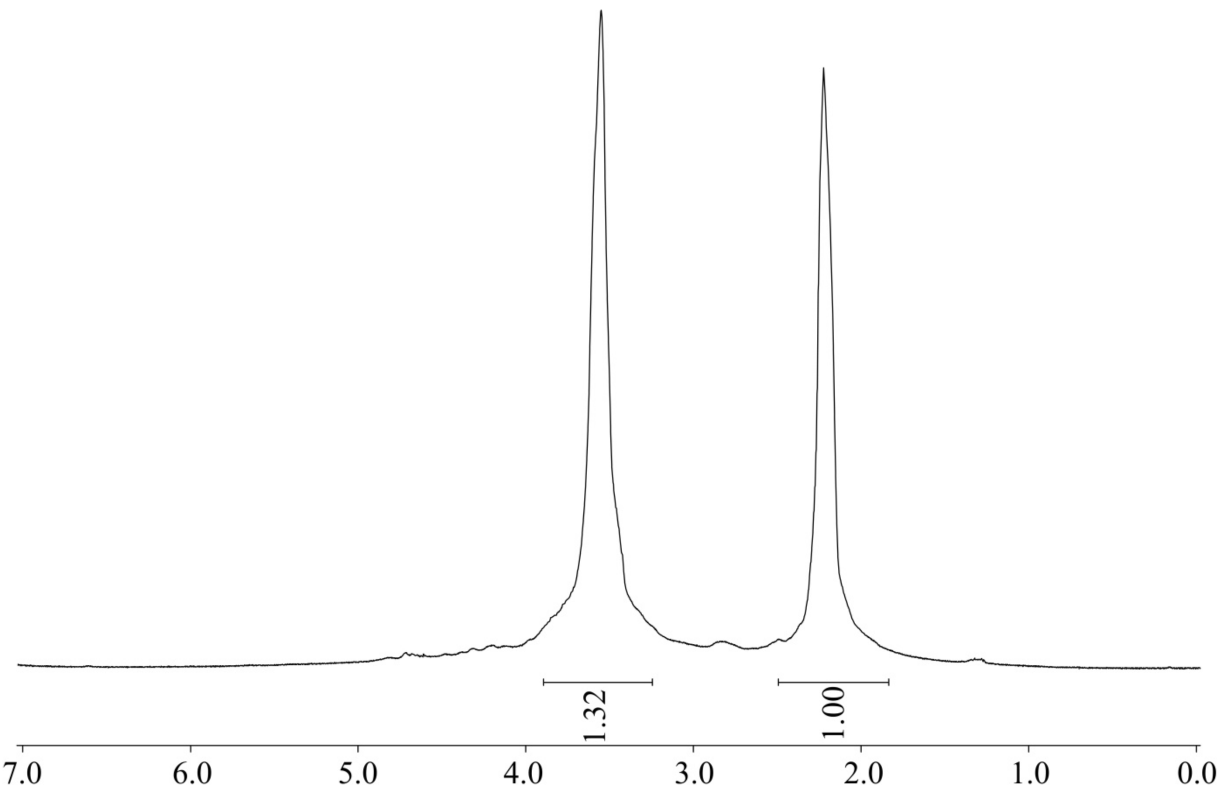

Fig. S3 – ^{1}H NMR spectrum poly(2-methyl-2-oxazoline).

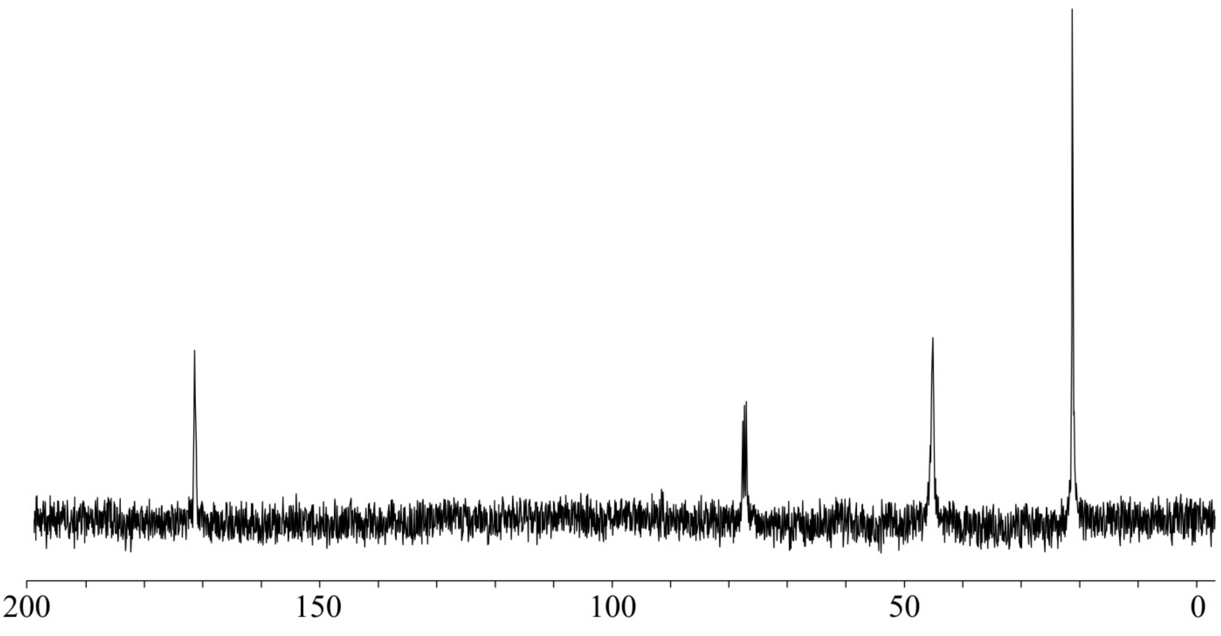

Fig. S4 – ^{13}C NMR spectrum poly(2-methyl-2-oxazoline).

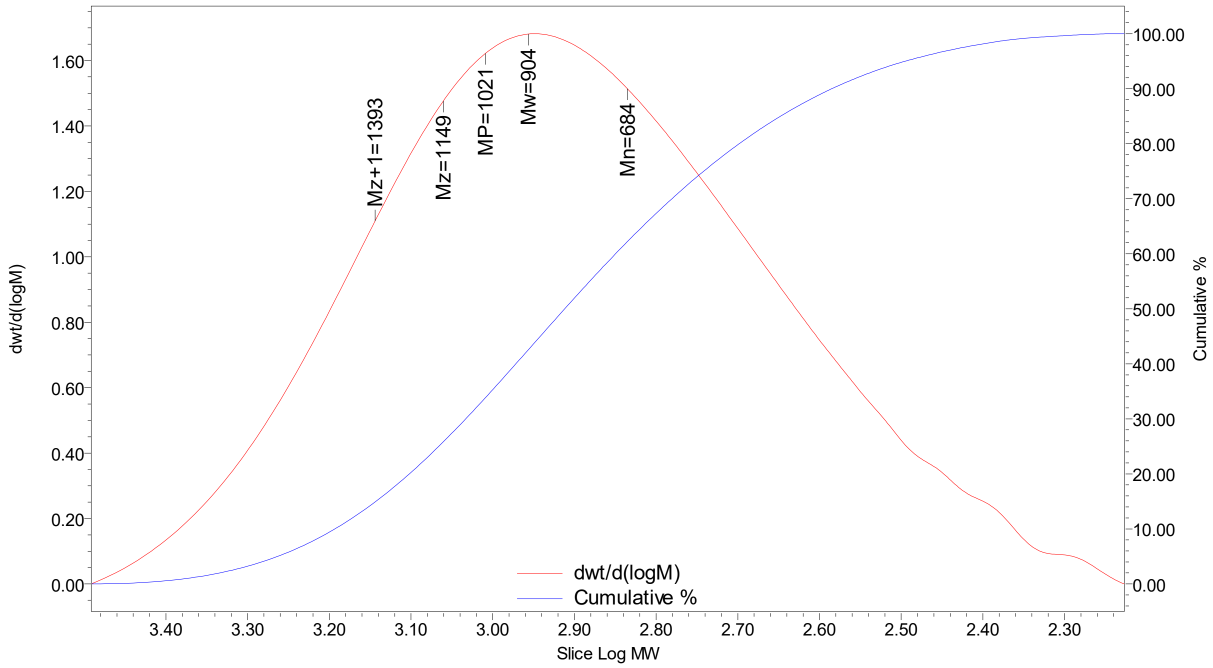

Fig. S5 – GPC curve of the synthesized poly(2-methyl-2-oxazoline).

Tab. S1 – Molecular weight characteristics of the synthesized poly(2-methyl-2-oxazoline).

Retention Time	Mn	Mw	MP	Mz	Mz+1	Polydispersity	Mz/Mw
14.804	680	900	1020	1150	1390	1.32	1.27

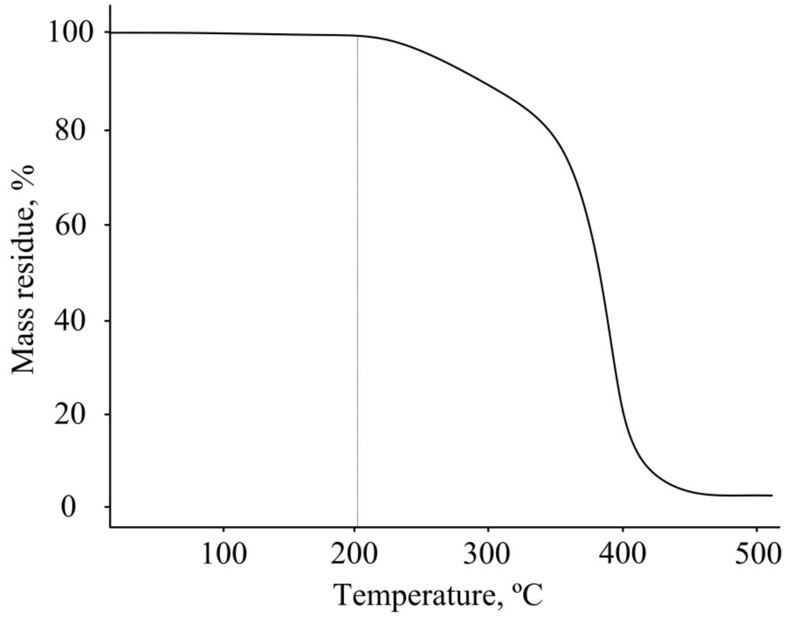

Fig. S6 – TGA curve poly(2-methyl-2-oxazoline).
